# Contrasting rule and machine learning based digital self triage systems in the USA

**DOI:** 10.1038/s41746-024-01367-3

**Published:** 2024-12-27

**Authors:** Bilal A. Naved, Yuan Luo

**Affiliations:** 1 Department of Biomedical Engineering, Northwestern University McCormick School of Engineering, Chicago, IL USA; 2 Department of Preventative Medicine, Northwestern University Feinberg School of Medicine, Chicago, IL USA

**Keywords:** Patient education, Machine learning, Computational models

## Abstract

Patient smart access and self-triage systems have been in development for decades. As of now, no LLM for processing self-reported patient data has been published by health systems. Many expert systems and computational models have been released to millions. This review is the first to summarize progress in the field including an analysis of the exact self-triage solutions available on the websites of 647 health systems in the USA.

## Introduction

**Fig. 1:**
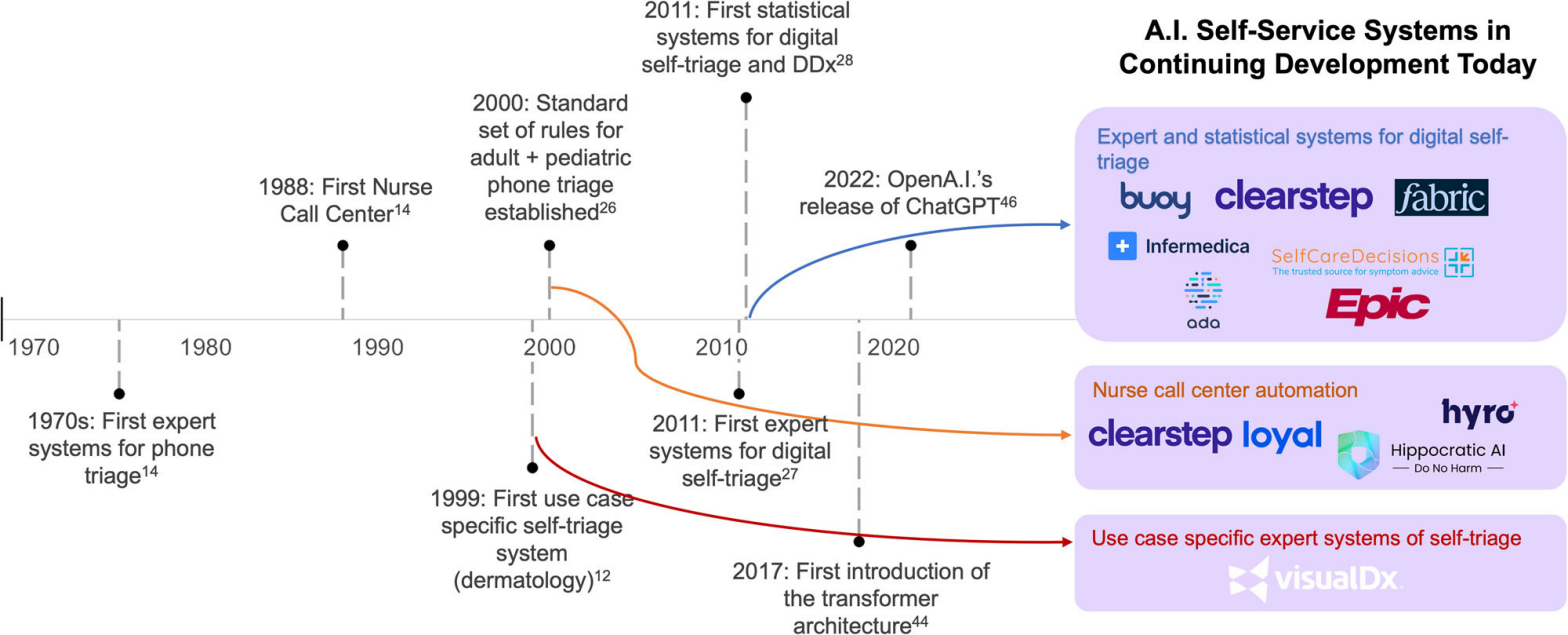
Timeline for developing artificially intelligent systems that use patient self-reported data. This timeline starts with the nurse call centers and shows how eventually their underlying content and decision rules were automated. Today there are a few distinct categories of patient self-service technologies powered by artificial intelligence.

Today, patients benefit from digital self-triage, automated intake, differential diagnostic, and disease characterization systems^1–12^. Prior to these automated systems, patients have had to navigate healthcare decision-making supported by the limits of their own knowledge, internet search browsers, and of people they contact, particularly medical professionals. Nurse call centers have been around for decades (Fig. 1). These services enabled patients to call a number, report their symptoms/conditions, and receive information to support their healthcare decisions (i.e., Patient Decision Support (PDS)). The most widely adopted telephone triage protocols are the Schmitt–Thompson telephone triage protocols (STPs) today powering 95% of nurse call centers across the country and used in >200 million encounters^13–15^. The STPs are a set of triage rules that help determine whether care is needed and if so, specifically which type of care. They resolve between emergency medical services (911), emergency rooms, urgent care centers, and PCPs. How many of us have been uncertain of which care to seek or whether to seek it at all when sick? The purpose of these STPs is to standardize and improve the accuracy of guidance medical professionals give over the phone in a textbook published by the American Academy of Pediatrics that continues to be maintained^14,15^. As nurse lines grew to be ubiquitously offered by every health insurer and health system, PDS grew to be robust^16,17^. As technological capabilities improved, automating these rules became an option and a simpler means for patients to self-support^18,19^.

These “virtual triage” systems, initially helping with intake and triage, were among the first mechanisms for patients to self-report their symptoms and receive PDS. However, automation came at a tradeoff. Increased speed, anonymity, and convenience came at the cost of a decreased ability to clarify and interpret responses via a medical professional on the phone^4,9,10,19,20^. On the other hand, the healthcare system could also save money by better optimizing use of their call centers^10^.

As data availability and technological capability improved, so too did computational statistical methods. Computers were programmed to do tasks typically reserved for human intelligence (Artificial Intelligence—AI)^21^. Eventually, methods were created that allowed computers to learn from large amounts of data and generalize such learnings to complete tasks (Machine Learning—ML)^22^. Combine these with the ability for computers to process natural language input by users (Natural Language Processing—NLP) and the building blocks for more powerful differential diagnostic engines and disease characterization abilities were set in place^23,24^. Eventually the transformer and large language models (LLMs) were developed leading to another inflection point^25–27^. Processing and generating natural language reached new heights with “generative A.I.” and automation no longer necessitated a tradeoff in ability to clarify or interpret patient-inputted language^28^. However, there are still gaps related to hallucination, interpretability, validation, and accuracy^21,29^.

Automated intake, virtual triage, and differential diagnostic prediction blossomed in industry and the start-up world^30–34^ enabled by the fact that the FDA has gone through multiple draft guideline revision periods but still has no enforced guidance on PDS systems^35,36^. This perspectives piece will dive deep into the nuance of self-reported virtual triage and differential diagnostic systems. It will discuss various approaches to building, validating, and improving on both. It will detail the advantages and disadvantages of rule- versus ML-based systems (Table 1). Finally, it will discuss the regulatory landscape and future directions.

**Table 1:** Advantages and disadvantages of expert systems and statistical learning methods for self-triage by health systems

		Expert based systems		Statistical machine learning	
		Advantages	Disadvantages	Advantages	Disadvantages
Important considerations of self-triage solutions for health systems	Validation	STPs have been tested in >200 million nurseline encounters over 20+ years^12,83^.	Requires a panel of physicians to iterate in determining the exact rules. There is no gold standard for self-triage. The next closest thing is telephone triage^84^.	Statistical triage systems use large datasets to train models (often a neural network) to calculate a triage level. The origin of these datasets is unknown in the literature^85,86^.	Statistical triage systems have been around for ~10 years in contrast to 20+ years for expert triage systems. Naturally, they are less validated^63,83^.
	Follows clinical best practice guidelines	The field of Emergency Medicine is centered around triage. Able to encode experiential knowledge gained in the field^18,87^.	There may be triage patterns that have not yet been uncovered by humans for encoding^88^.	Potential to identify triage patterns that optimize for minimal resource utilization and maximal likelihood of symptom resolution^18,89–92^.	Triage does not have a "gold-standard". So there is no "right answer" with which to label training data^93^.
	Improvement over time	Systems may have their own rules for triage that vary based on available resources in facilities. Expert systems allow for this configurability^94^.	Improvement over time requires random sampling of real triage encounters and human reinforcement. Also requires paying attention to triage distributions at a population level and adjusting as needed^95^.	If provided with feedback on the correctness of an individual patient’s triage, machine learning-based systems may enable more automated improvement over time^96,97^.	There is no standard set of "triage-specific" data in the EMR. Just because an individual patient went to X location, does not mean it was the "right" location for their care. The EMR does not contain direct information on whether or not that site of care was "right" or if another one would have been more appropriate^98–109^.
	Explainability	Any prediction may be examined for the exact rules that led to it and adjusted as needed^110^.			If an issue with triage is found, it is difficult to understand exactly why that recommendation was provided and to adjust that specific pathway^111^.
	Length	Asks less questions overall (avg: 17.5)^63^.			Asks more questions (avg: 29.2)^63^.

## Triage

### Definition

The medical term for triage comes from the French verb, *trier*, meaning to sort or separate^37,38^. Accordingly, triage is a process of assigning priority to a patients’ treatments based on the severity of their condition. This process helps allocate limited medical resources and is used in a variety of settings^37^. After its development and use in wartime settings, triage was then generalized for emergency settings in civilian life^39^. Triage is commonly used in emergency departments to determine the order in which patients are seen^40^. More generally, primary care providers (PCPs) must triage which patients can be managed by them versus which patients must be managed by specialists. While individual hospitals or systems may develop their own rules to support PCPs decision-making, there is no standardized set of clinical guidelines that determine exactly which patients should be referred to specialists^41^. Today, patients must decide to access care among a growing list of emergency services, emergency rooms, urgent care centers, walk in/retail clinics, telemedicine providers, primary care offices, specialist offices, and more. As a result, 20–40% of healthcare spend is wasted each year in unnecessary healthcare utilization^42^. To minimize the usage of resources there is a need to enable patients to better self-triage. Patient-facing digital technology has made it possible for PDS to be provided in patient-friendly formats. There are generally two classes of virtual triage systems: (1) rule-based systems and (2) machine learning-based systems. Each of these two classes offers certain advantages and disadvantages that will be overviewed in the following sub-sections.

### Rule-based self-triage systems

Rule-based virtual triage systems employ a set of empirical rules, designed by medical professionals, to guide patients with a specific set of signs, symptoms, and conditions to the most appropriate site of care within the most appropriate timeframe. Individual hospitals or health systems may devise their own rules to help PCPs determine which patients should be referred to which specialists^41,43^. However, the first nationally adopted set of triage rules were for telephone triage^44^. Most health systems in the USA trust the rule-based protocols developed by Drs. Barton Schmitt and David Thompson for telephone triage of patients (STPs)^45^. Rules-based systems serve to encode medical knowledge and experience accumulated over the lifespan of clinical medicine and allow for complete configurability and explainability of any triage. The STPs have been used in >200 million nurse call center encounters^45^. The disadvantages of rules-based systems are that they are laborious to create, require a reconciliation process between multiple physicians complicated by the fact there exists no triage gold standard and there is inherent variation among how different medical professionals might triage patients, and that triage accuracy would be limited to the accuracy of the humans developing the rules^46^. Some solutions have conducted this reconciliation process to better standardize the exact rules that would indicate which patients should see a specialist versus a PCP (Table 2). These solutions use decision trees or knowledge graphs to power self-triage experiences that are encoded with clinical best practice guidelines as they’ve been developed and validated to date (e.g., STPs).

**Table 2:** A description of each self-triage vendor currently in use today by health systems across the USA

Name	First self-triage or DDx?	Type	Description	Source
Clearstep	Triage	Hybrid	Expert system built off Schmitt triage content (co-author of the Schmitt–Thompson telephone triage protocols) + Hybrid supervised and large language computational statistical model for natural language processing and output of free text conversationally and for summarization of the experience.	Industry knowledge
Fabric (fka Gyant)	Triage	Statistical	Uses NLP to extract SNOMED, ICD 10, and its own concepts out of EHR data. All this information is structured and labeled by Gyant clinicians. This includes adjusting for prevalence differences. A random forest model is then trained using the labeled, structured data.	^112^
Self-care decisions	Triage	Expert system	List-based checklist of decision rules that the patient goes down sequentially and rules out one at a time. The first rule unable to be removed from the list is chosen for its triage disposition. Based on Schmitt clinical content (co-author of the Schmitt–Thompson triage protocols).	^113^
MSFT health bot	Triage	Depends on triage source	Set up to be powered by any virtual triage vendor. Currently uses infermedica in its backend.	^114^
Ada	DDx	Hybrid	Medical Knowledge Base: Ada hosts a medical knowledge base, which is used to define a Bayesian network. The knowledge base was built and reviewed by medical doctors in a curated process of knowledge integration from medical literature and is continuously expanded. The knowledge base consists of disease models of common conditions and several hundred rare diseases, including their corresponding symptoms and clinical findings, which are added to / updated in the knowledge base according to evidence from peer-reviewed literature. Components of the knowledge base can have additional metadata associated with them and used for further refinement (e.g., intensity, temporality). Epidemiologic data is used to define probabilities of disease at baseline prior to any responses provided by the user. Question Selection: Each question asked by Ada is dynamically and probabilistically determined by the reasoning engine. Each subsequent question is determined based on all previously supplied basic health information and symptoms. The engine is designed to balance the number of questions asked with differential diagnostic rigor. Reasoning Engine: Ada’s reasoning engine estimates disease probability using the medical knowledge base via a Bayesian network that carries out approximate inference on the base. “Information-theoretical methods” are used to decide which questions are asked to the user. Validation + QA/CE: Ada’s medical intelligence (i.e., reasoning engine + medical knowledge base) is continually validated against a set of several thousand internal test cases. These test cases are made of common and rare diseases from across medical specialties and includes cases based on medical literature as well as “bread-and-butter” case scenarios of varying levels of diagnostic certainty. A team of medical doctors employed by Ada constantly review the system’s medical knowledge. A second process involves a verification tool to test each update to the medical intelligence with hundreds of cases written by doctors external to Ada. Ada medical doctors are blinded to the second set of test cases, which is also regularly updated.	^115^
Bright.MD	Triage	Expert system	The three health system implementations using BrightMD’s technology offer no questions to calculate a triage. The experience includes a keyword search, proceeded by a list of symptoms indicating that emergent care is needed for the chosen chief complaint, after which the patient is shown options for care if they click “next” (i.e., endorsing they do not have any of the emergent symptoms)	^116^
Healthwise	Triage	Expert system	Expert system that goes through a series of pre-determined questions that has the ability to branch based on prior responses and is built off Healthwise clinical content.	^117^
Orbita	Triage	Hybrid	Uses Isabel for self-triage but then transforms Isabel’s content into conversational A.I. via Orbita’s technology.	^118^
Isabel self-triage	Triage	Expert system	The Isabel Self-Triage asks 7 standard questions for any chief complaint and calculates pre-programmed triage dispositions among 4 different levels of acuity.	^119^
Buoy	DDx	Statistical	“An undisclosed algorithm, supposedly relying on natural language processing (NLP)–extracted data from 18,000 clinical papers. As stated by its chief executive officer and founder, Buoy Health specifically does not use decision trees but “dynamically picks” 1 of 30,000 questions based on the principle of greatest reduction of diagnostic uncertainty, which does not necessarily imply the use of neural networks”.	^120^
Infermedica/Symptomate	DDx	Statistical	1. Collecting initial evidence. The patient interview starts with gathering initial symptoms, risk factors, and demographic data. Additional input regarding the symptoms’ occurrence or severity makes the engine even more precise. 2. Intelligent interview. The inference engine uses initial evidence to construct a dynamic interview based on probabilistic models and reasoning techniques. The algorithms follow the rules of differential diagnosis that physicians use to interview the patient. The engine evaluates various conditions at once. 3. Triage recommendations based off a differential. Finally, the inference engine presents the most probable causes of the symptoms, which are paired with the suggested level of care. Triage recommendations are based on a 5-level scale (self-care, consultation, consultation 24 h, emergency, ambulance).	^121^

Inclusion criteria for this table is the same as for Table 2 and the order of solutions described follows that of Table 2 as well.

### Machine learning (ML)-based self-triage systems

In contrast to the rule-based systems that use empirical decision rules, an ML-based approach to virtual triage depends on training a model on a large set of patient data to predict the most appropriate site of care for that individual to go to. With many of the statistical approaches it is often difficult to provide the exact patient responses that led to their triage result (e.g., neural networks, deep learning, transformer-based)^47^. This impacts the explainability and interpretability of statistical machine-learning (ML) approaches. Certain statistical approaches may allow for some explainability (e.g., non-neural network approaches like regression models and tree-based feature importance)^48,49^. These are typical limitations of ML-based approaches at large.

One of the challenges in labeling datasets for supervised learning is that it may be difficult to establish a “right-answer” for triage. This is because different healthcare providers (HCPs) would triage certain patients differently^46^. As a result, the available training data is subject to variability. Additionally, there is no consensus on the basis by which to conclude that a patient was “successfully” triaged. Numerous sites of healthcare delivery could resolve a patient’s issue (Box 1). Theoretically, perfectly accurate self-triage would always point the patient to the optimal site of care at the least cost. Conversely, inaccurate self-triage would lead to more unnecessary touchpoints and greater cost. The current best practice in this field of triage are the rules trusted in nurse call centers for the past few decades (STPs). Success metrics could include the number of visits required to resolve/manage a patient’s medical concern or the cost of care delivered to the patients (Box 1). These two metrics could be combined into a ratio. We suggest this ratio with the idea that “successful” healthcare would ideally resolve a patient’s medical concern using the minimum healthcare resources to do so. Using some defined measure of success, machine learning models could be trained to recommend certain levels and timing of care. As an example, we could create a model that minimizes referrals to other care and utilization of healthcare resources.

Despite the disadvantages of ML-based approaches to virtual triage, there are several advantages. It can be both less time intensive and costly to maintain a machine learning model once the training, testing, validation, and operational pipelines have been established. However, choice of labels is important. Data labeled manually by humans will results in models that can only achieve parity with human decision-making. Training models to achieve certain outcomes (maximizing the ratio of problem resolution to cost) may reveal patterns that humans would not have recognized. A hybrid approach is hypothesized to blend human experience with added nuance. Additionally, an AI-approach would theoretically be able to capture patterns that humans cannot/have not yet. Moreover, as more training data is provided to the model, it can continuously get better over time. This process of continuous improvement can also be automated.

Box. 1 This scenario shows a sampling of pathways that one patient may take to receiving the same overall care as a function of different “first touchpoints”. All clinical pathways are displayed after expert consultation with PCPs and specialists **Illustrating the impact of the first touchpoint on the overall care journey for a single patient**

Anonymized patient scenario*: 30 y/o in Chicago with knee instability that started after playing basketball aggressively. It was not preceded by any inciting event or contact. The patient didn’t feel a “pop”. There is no pain, swelling, or redness. The knee doesn’t give out. The instability almost disappears with rest but gets worse after exercise.

Clinical guideline: In states like Illinois where physical therapy (PT) can refer to imaging: referral to PT and referral to radiology for an X-ray. If no improvement after PT, they can refer for an MRI with potential referral to Orthopedics after depending on the findings.

First touchpoint options: In-person PCP, virtual PCP, specialist visit (PT and/or orthopedics), urgent care, asynchronous care. For this illustration, we assume this patient did not get better with PT and thereby required MRI imaging, which revealed the need for an orthopedic consultation.

**Table:** 

Sample patient pathways leading to same overall care administration					
First touchpoint	Second touchpoint	Third touchpoint	Fourth touchpoint	Overall number of steps	Bill Amount (pre-insurance)**
In-person PCP	PT	Radiology	Orthopedics	4	~1400
Virtual PCP	PT	Radiology	Orthopedics	4	~1300
PT	Radiology	Orthopedics		3	~1250
Orthopedics	PT	Radiology	Orthopedics	4	~1500
Urgent care	PT	Orthopedics		3	~1350

Traditional Care Pathway (without using self-triage): Start with PCP or Orthopedics (4 steps + $$$)

Self-triage (minimize # of touchpoints + cost): Start with PT (3 steps + $$)

*Note: this is a real patient scenario included anonymously and with consent

**Bill amount estimated using the following references^122–124^

## The use of large language models for self-triage

November 2022 marked an inflection point in what was possible with A.I with the release of ChatGPT and a subsequent wave of LLM proliferation ensuing across industries. LLMs’ ability to process and convert inputs into engaging, human-like outputs marked an inflection point in what was possible with automated conversational experiences with patients having reported the desire to use LLMs for healthcare-related inquiries^50^.

Thus far, LLMs have been evaluated for their rapport with patients, their triage and DDx accuracy in general, and their utility for patient self-education and self-diagnosis in a collection of specific use cases (e.g., COVID^51^, ophthalmology^52^, obstructive sleep-apnea^53^, sialendoscopy^54^). However, the commercial use of LLMs in healthcare has been largely limited to clinical documentation and other administrative functions on the provider-facing side. The patient-facing side has yet to demonstrate the same level of adoption.

Much of the slowness in adoption on the patient-facing side has been due to a list of concerns with LLMs. Including, but not limited to, hallucination rates, consistency of response, interpretability, regulation, and copyright^55^. ChatGPT was found to be 63% accurate on average in 10 different reasoning categories^56^. In healthcare, the risk of an incorrect recommendation comes at a cost to the patient’s health. Thus, it is imperative that patients and healthcare professionals have the capability to interpret the exact reasons for a model’s prediction/recommendation. In general, this is a drawback of ML approaches to patient-self reported triage or diagnosis^57^.

Considerable work is being done to evaluate the full scope of LLMs’ viability in healthcare. Levine et al. compared the triage and diagnostic accuracy of GPT-3 to a standardized set of vignettes used historically for evaluating the accuracy of self-triage systems. GPT-3 performed diagnosis at levels close to, but below that of physicians and better than that of lay individuals. The model performed less well on triage, where its performance was closer to that of lay individuals and below that of clinicians^58^. Hirosawa et al. evaluated the diagnostic accuracy of differential diagnoses generated by GPT-3 on a collection of commonly reported chief-complaints and found GPT-3 to be 40% less accurate on average compared to the judgment of physicians^59^. Chiesa-Estomba et al. evaluated the potential of GPT-3 as a supportive tool for sialendoscopy clinical decision making and patient information support. GPT-3 was found to have strong concordance with ENT specialists^54^. When responses to patient questions from GPT-3 were compared to those from physicians, patients reported that responses from the LLM were 10× more empathetic than those from physicians^60^. Moreover, surveyed patients indicated the desire to use LLMs for healthcare-related inquiries^50^. All in all, LLMs have significant progress to make in achieving comparable accuracy to physicians in self-triage and self-diagnosis use cases, have demonstrated clear demand from patients, and have outperformed physicians in the empathy of their responses. Regulation aside, there is clear potential for using LLMs in the spaces of self-triage and self-diagnosis.

## FDA regulatory landscape

Currently, decision support systems that make use of patient self-reported data are not regulated by the FDA. However, the FDA has released several iterations of draft guidelines which have been open for public commenting^36^. Through these iterations there have been several developments. Initially, the draft guidelines separated PDS and clinical decision support systems (CDS) as two different categories^61^. However, these two categories have now been merged into one broader category of CDS^36^. CDS are those that fit the following four criteria: The system is “(1) not intended to acquire, process, or analyze a medical image or a signal from an in vitro diagnostic device or a pattern or signal from a signal acquisition system (2) intended for the purpose of displaying, analyzing, or printing medical information about a patient or other medical information (3) is intended for the purpose of supporting or providing recommendations to a HCP about prevention, diagnosis, or treatment of a disease or condition and (4) is intended for the purpose of enabling an HCP to independently review the basis for the recommendations that such software presents so that it is not the intent that the HCP rely primarily on any of such recommendations to make a clinical diagnosis or treatment decision regarding an individual patient^36^.

Systems that fit these four criteria are exempt from regulation by the FDA. Note that the last of these criteria has important implications on the architecture of successful triage and DDx systems. To enable patients to see the basis by which information is recommended to them is more difficult for systems that operate off an ML approach as compared to ones that are rules-based. If the specific ML approach is unable to fit this fourth criteria, then it would not be exempt from FDA regulation and would be subject to further scrutiny. This would require greater resources to successfully bring ML-based systems for generalized use with patients. Should the draft FDA clinical decision support guidelines become law, rules-based or hybrid systems may have an advantage from a regulatory perspective. A limitation of this review is its focus on the U.S. healthcare system. It does not include a regulatory overview of other regions around the world which may be the subject of future work.

## Challenges and future directions

The generalizability of AI to clinical practice remains in question^62^. Many different ML models have been reported in the literature for their ability to outperform humans in medical settings, however, few have been generalized for broad, clinical use^62^. Often, these models do not perform as strongly when generalized to the population at large. We hypothesize that the most successful approaches will employ a hybrid methodology of empirical knowledge and statistical methods. Simply stated, using the centuries of human medical experience to encode rules that are generally agreed upon by most medical experts would likely enable systems to achieve comparable levels of accuracy to human clinicians. Then building statistical layers on top of these empirical rules would enable systems to advance accuracy beyond that of human ability and simultaneously enabling more conversational experiences.

There is limited published data on the performance of self-triage systems. The seminal paper comparing the accuracy of various knowledge-based and statistical triage and diagnostic systems found, that among 23 symptom checkers, the correct diagnosis was provided first in 34% (CI: 3%) of a set of standardized patient vignettes, in the top 20 diagnoses in 58% (CI: 3%) of vignettes, and the appropriate triage in 57% (CI: 5%) of vignettes^63^. A subsequent study comparing 37 systems reported similar results^64^. Triage performance decreased with decreasing urgency of condition^63^. The accuracy of triage systems for emergent scenarios was 2–3× higher than in self-care scenarios^63^. Moreover, accuracy ranged widely as performance on appropriate triage advice from 24 symptom checkers varied from 33 to 90%^63–65^.

This wide variation in accuracy of self-triage systems reveals one of its limitations. It is possible that *inaccurate* self-triage increases the total touchpoints and cost. To illustrate this point, one can conduct a thought experiment with the following groups: (a) users of a “dummy” self-triage system that randomly assigns a triage to each patient and (b) users of a “perfect” self-triage system that is able to perfectly optimize all factors of clinical accuracy, insurance, patient preferences, provider preferences, and network preferences. One hypothesis is that users of (a) are bounced around the system more often. Potentially until they get lucky to be with the right provider for their need. In contrast users of (b) would always be presented with the exact provider for their immediate clinical need, thereby minimizing the number of touchpoints, cost, and time expended to receive that optimal care. The effect of self-triage systems on average # of patient touchpoints at the health system, average cost of overall care, or on clinical outcomes is yet to be reported on. Regardless, any study that examines these outcomes of self-triage will also have to account for variation in accuracy based on the individual triage system used.

One gold standard of triage accuracy is the judgment of human clinicians. There are studies assessing concordance of self-triage and diagnostic systems with medical professionals. Several systems with rules- and ML-based layers have been found to perform at least as well as doctors^46,66^. As more statistical methods are tested, self-triage and diagnostic systems continue to improve. One group hypothesized that physicians spend more time ruling out than in. Thus, a counterfactual system was found to be more accurate than an inferential one^67^. Moreover, as greater connectivity between these systems and the electronic medical record (EMR) are created, feedback will increase. Self-triage systems can begin to optimize for minimal referrals and cost while maximizing for resolution and satisfaction. Hybrid rules- and ML-based systems are emerging^46^. Eventually, integration with wearable and sensor technology will facilitate the incorporation of objective information about a person’s health status further improving accuracy. Understanding the tradeoffs between various rules- and ML-based methods will be critical to designing successful self-guided PDS systems. Moreover, navigating the emerging regulatory landscape will also determine success. User experience (UX) research and design to optimize the manner in which patient users interact with the self-triage systems will be critical. Considering self-triage systems require significant amounts of information (tens of questions) to be answered by the patient users, there is a high percentage of drop-off. Optimizing the UX for ease and maximal completion represents an emerging body of literature.

One of the most common modalities for administering self-triage systems is via a chatbot^6,9,63,68^. Research into the design and optimization of healthcare chatbots has identified several key insights to enhance user engagement and minimize drop-off rates. In healthcare settings, the perception of empathy and understanding from a chatbot can make users feel more comfortable^69^. Furthermore, research highlights the importance of fairness in chatbot responses. One study indicates that users are sensitive to perceived biases or unfairness in chatbot interactions^70^. Ensuring transparency and fairness in chatbot behavior directly correlates with higher satisfaction and trust, underscoring the need for ethical design principles in UX development for healthcare systems^71^. In addition, the visual presentation and conversational style of chatbots can also influence how users perceive and engage with the system, though these factors may not always directly improve user experience without thoughtful integration^69^.

To reduce the drop-off rates in self-triage systems, best practices from chatbot research suggest focusing on creating seamless, intuitive interactions that reduce cognitive load while still collecting essential information^72^. This includes optimizing the length and complexity of questions and designing conversational agents that can adapt to user responses dynamically, keeping the interaction engaging and efficient^70^. The potential for more effective and human-centered designs lies in balancing these technological and psychological factors to create an experience that feels personalized and responsive.

To ensure the experience also feels safe, a number of ethical and privacy issues must be considered. First, protecting patient privacy is paramount. These systems collect and analyze sensitive health data, and thus must comply with strict data security standards. These include the prevention of storing identifiable health information, automated data wipes, auditing of organizational processes and data, and the certification of upholding strict security standards (e.g., HIPAA, SOCII, or HITRUST). Additionally, users should have control over how their information is gathered and used, ensuring informed consent and autonomy in healthcare decisions^73^. Equally important is the need to ensure that self-triage systems operate equitably. These tools must be designed to account for systemic inequalities, such as those faced by individuals with disabilities or those from disadvantaged socioeconomic backgrounds. Incorporating equity adjustments, like reserving care for marginalized groups, helps to mitigate biases in algorithmic decision-making^74,75^.

Transparency is another critical ethical consideration. Providing patient users and HCPs the ability to understand the logic behind decisions made by self-triage algorithms builds trust and ensures proper use^76^. Doing so also aligns with the draft guidance from the FDA^36^. Without clear and accessible information about how these systems function, there is a risk of mistrust or misuse, potentially leading to harmful outcomes for patients^77^. Finally, while self-triage systems can improve efficiency, healthcare professionals must be able to intervene when necessary to correct algorithmic errors or biases, ensuring that critical health decisions are not left entirely to automated systems^74,78^.

Addressing these ethical issues—privacy, equity, transparency, and human oversight—will allow self-triage systems to contribute positively to healthcare delivery, without exacerbating existing inequities or undermining user trust. The intersection of self-reported patient data, artificial intelligence, and user experience research in the form of self-triage systems, represents an important step towards more proactive, automated, optimized, and self-guided healthcare delivery.

But to achieve this future will likely require deeper collaboration between industry partners including the electronic health record companies, community health systems, payers, and technology companies. This is because self-triage systems’ accuracy is limited without a “right answer” to affirm or deny the validity of a recommendation. However, without access to the data found in the EMR or in claims there is no way to “close the loop” for validation purposes. This unmet need in the field results in slower progress to improving the patient’s navigational experience of healthcare. Collaborations between these partners would solve for this. While medical centers house the majority of data that would be required to validate such systems, the majority of innovation in A.I. self-triage systems has been in the private industry (Table 3).

**Table 3:** Health system adoption of virtual triage solutions by vendor on websites accessible to the public (unauthenticated)

Virtual triage solution adopted by regional US health system(s)	Count	%
No adopted virtual triage solution	598	92.42%
Clearstep	13	2.01%
Fabric (fka Gyant)	10	1.55%
Self-care decisions	6	0.93%
MSFT (COVID)	6	0.93%
Ada	4	0.62%
BrightMD	4	0.62%
Healthwise	2	0.31%
Orbita	1	0.15%
Isabel	1	0.15%
Azure Healthbot (MSFT)	1	0.15%
Buoy	1	0.15%
Grand Total	647	100.00%

Due to the novelty of virtual triage 92.42% of health systems have not yet adopted any publicly accessible solution. Of the remaining 7.57% that have adopted a solution (49 systems), 12.24% of them have only adopted COVID-specific self-triage (6 systems).

## Health system adoption of self-triage systems

As part of this review, self-triage adoption by health systems in the USA was measured (Table 3 and 4). The websites of 647 health systems were examined by applying the following inclusion criteria from a complete list of U.S. hospitals and health systems^79^: (a) the system owns at least 3 hospitals and generates at least $250 M in net patient revenue, (b) the system has a distinct web experience, and (c) the self-triage experience is available to the public. Out of this list, 15 different self-triage systems were found to be adopted by 50 health systems (7.72%) with 6 of those (0.93%) being self-triage exclusively for COVID. Of the 15 self-triage systems, the most widely adopted vendor was available on the websites of 13 health systems (2.01%) with the next highest being available on 10 health systems (1.55%) each (Table 3). As US health systems vary in size, the number of hospitals serviced by each health system was also incorporated into the analysis. Table 4 shows that 534 hospitals (8.73% out of a total of 6120 in the USA^80^) have websites with self-triage readily available. The most widely adopted vendor was available on the websites representing 244 hospitals (3.99%). The next highest being available on the websites representing 91 hospitals (1.49%). A future study could examine the differences in routing needs required for technology to optimize patient access with the delivery of healthcare resources at varying scales of service area (e.g., 100 hospitals vs 300 hospitals). Additionally, more details on each system are provided in Table 2.

**Table 4:** Hospital adoption of virtual triage solutions by vendor on websites accessible to the public (unauthenticated)

Virtual triage solution in use by number of regional US hospital(s) covered	Count	%
No adopted virtual triage solution	6136	92.21%
Clearstep	244	3.96%
Fabric (fka Gyant)	91	1.36%
MSFT (COVID)	42	0.63%
BrightMD	30	0.45%
Buoy	30	0.45%
Healthwise	21	0.31%
Self-care decisions	20	0.30%
Ada	17	0.25%
Isabel	11	0.16%
Orbita	9	0.13%
Azure Healthbot (MSFT)	3	0.04%
Grand total	6654	100.00%

92.21% of hospital websites do not provide patients with public access to self-triage. Of the remaining 7.79% that have website access to a solution (518 hospitals), 8.10% of them have only adopted COVID-specific self-triage (42 hospitals).

For now, the majority of patients who are experiencing a new symptom must self-triage to an appropriate site of care. As a result, one study shows that 60% of the time patients inappropriately triage themselves^81^. At scale, it is estimated that almost a trillion dollars of healthcare expenditure (i.e., 25% of total health care spending) is wasted^82^. Self-triage systems hold great promise in supporting patients to make more clinically accurate decisions. In fact, the same study showed that 15–30% of patients who used a digital self-triage system engaged with triage results for re-direction to more clinically appropriate care^81^.

As health systems, payers, EHR companies, and self-triage companies work together more, partnerships between them would benefit patients by providing a “closed loop” of feedback to A.I. systems. Doing so would enable such systems to validate predictions and learn more quickly with increased use; ultimately providing more intelligent navigational capabilities. As a result, less patients would bounce around the health system and more would likely seek the right care at the right place and right time. Additionally, doing so would also benefit payers as more efficient healthcare utilization would lead to reduced cost of care per patient. Finally, providers could expect to see more patients that are appropriate for their them. This is especially relevant in the context of current provider shortages and burnout. More than ever the healthcare system needs to ensure it is not wasting unnecessary resources. Enabling patients to better route themselves is a key component to controlling spend. There is a future in sight where the knowledge required for clinically accurate decision-making is democratized and made accessible to patients in a delightful experience that benefits everyday people regardless of their education level.
